# Recent Advances on IoT-Assisted Wearable Sensor Systems for Healthcare Monitoring

**DOI:** 10.3390/bios11100372

**Published:** 2021-10-04

**Authors:** Shwetank Dattatraya Mamdiwar, Akshith R, Zainab Shakruwala, Utkarsh Chadha, Kathiravan Srinivasan, Chuan-Yu Chang

**Affiliations:** 1 School of Electronics Engineering, Vellore Institute of Technology (VIT), Vellore 632014, India; shwetank.mamidwar2018@vitstudent.ac.in (S.D.M.); akshith.r2018@vitstudent.ac.in (A.R.); zainab.shakruwala2018@vitstudent.ac.in (Z.S.); 2 Department of Manufacturing Engineering, School of Mechanical Engineering, Vellore Institute of Technology (VIT), Vellore 632014, India; utkarshchadha1302@gmail.com; 3 School of Computer Science and Engineering, Vellore Institute of Technology (VIT), Vellore 632014, India; kathiravan.srinivasan@vit.ac.in; 4 Department of Computer Science and Information Engineering, National Yunlin University of Science and Technology, Yunlin 64002, Taiwan

**Keywords:** Internet of Things, healthcare, sensors, wearable devices, cloud systems, healthcare monitoring, data processing

## Abstract

IoT has played an essential role in many industries over the last few decades. Recent advancements in the healthcare industry have made it possible to make healthcare accessible to more people and improve their overall health. The next step in healthcare is to integrate it with IoT-assisted wearable sensor systems seamlessly. This review rigorously discusses the various IoT architectures, different methods of data processing, transfer, and computing paradigms. It compiles various communication technologies and the devices commonly used in IoT-assisted wearable sensor systems and deals with its various applications in healthcare and their advantages to the world. A comparative analysis of all the wearable technology in healthcare is also discussed with tabulation of various research and technology. This review also analyses all the problems commonly faced in IoT-assisted wearable sensor systems and the specific issues that need to be tackled to optimize these systems in healthcare and describes the various future implementations that can be made to the architecture and the technology to improve the healthcare industry.

## 1. Introduction

In a rapidly evolving world, it has become necessary for most, if not all, technologies to become interconnected, remotely accessible, and analyzable. To achieve this, we made use of Internet of Things. Internet of Things is a means of connecting devices to the internet, hence making such devices ‘smart’, e.g., smart watches, smart lighting, etc. IoT expands the independence of humans to interact, contribute, and collaborate with things [1]. IoT has been used in various fields such as agriculture, home automation, traffic management, delivery management, water supply management, fleet management, smart grid, energy saving, etc. [2].

IoT-assisted wearable sensor systems technology is a booming and blooming field in healthcare. As the healthcare sector expands, we need a doorstep diagnosis, easily monitoring and controlling the data. The end goal is to embed IoT in emergency services, connected homes, smart hospitals, EHR, etc. [3]. These data that we collect through intelligent devices and an intelligent hospital can then monitor patients’ symptoms in real-time. This can help us make discoveries regarding healthcare, medicine, drugs, and vaccines. The end goal of this is to make the data secure and accessible by the right people via cloud computing, fog computing, etc. [4].

**Figure 1:**
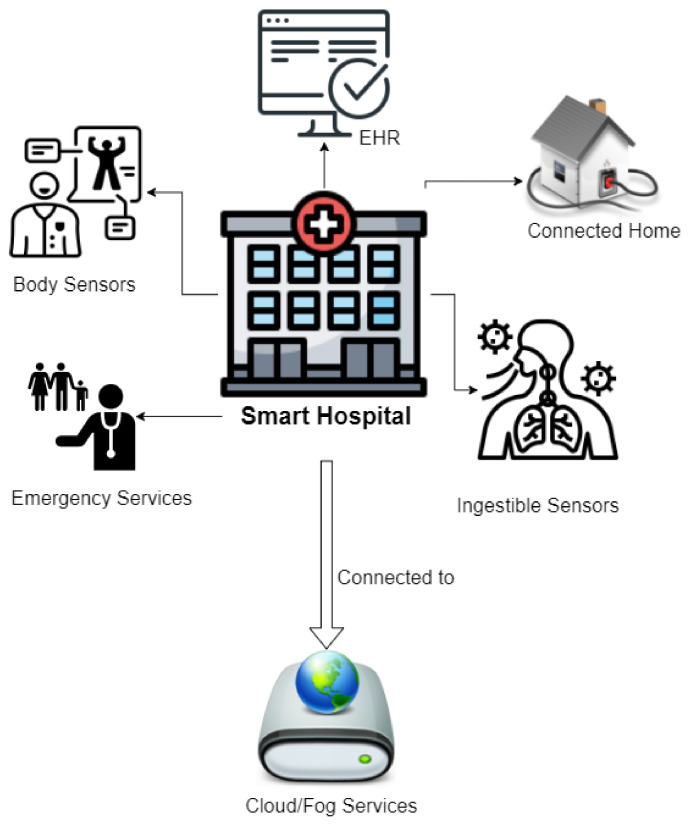
An outlook of IoT-assisted hospitals for healthcare monitoring.

For ensuring a quick and seamless transfer of data, different wireless technologies and communication protocols are considered. The data can also be used for analysis using data analytics. A WSN is a sensor network that is connected wirelessly with the help of different communication protocols. This review compares the brief overview of different deep learning algorithms and the WSN used to analyze and strengthen IoT in healthcare [5,6]. An EHR is a digital record of a patients’ paper chart. As shown in Figure 1, a smart hospital is built by body sensors, ingestible sensors, EHR, emergency services, remote monitoring, etc. [6,7]. This can be connected to cloud platforms via different communication protocols. Creating and managing a WSN requires wearable sensors and different healthcare monitoring systems. Merging all these elements helps us achieve smart healthcare [8,9].

### 1.1. Contribution of this Survey

This survey compares over 133 papers taken from esteemed journals. Research papers and review articles on IoT-assisted wearable sensor systems in healthcare have been taken into account. Papers on the different communication technologies in the field were also referred to. This survey provides a comprehensive study of the IoT-assisted wearable sensor systems in healthcare and can be a reference for further research. In Table 1, a brief comparison with previous surveys is given. Various papers were compared, and conclusions were drawn from them to reach a census and suggest possibilities for future improvement.

### 1.2. Survey Structure

This survey is based on more than 100 research works. In Section 1, a brief overview of the paper is given. The selection criteria of papers are discussed, and briefly, the various surveys are compared. In Section 2, the architecture of an IoT-assisted wearable sensor system for healthcare monitoring is described. A brief overview of the data cycle is given, and each stage is described—a detailed overview of the different wireless technologies used for communication in a WSN. In Section 3, the applications of IoT-assisted wearable sensor systems in healthcare applications are discussed. In Section 4, the wearable sensors used for healthcare monitoring are described. In Section 5, a brief overview of the current trends in the healthcare industry and a brief overview of FDA and CE approvals needed for the industry are given. In Section 6, the open problems and future opportunities in this field are given. In Section 7, the paper is concluded and lists the references toward the end.

### 1.3. Paper Selection Criterion

The paper rigorously tries to explain the need and application of the wearable device in the current healthcare domain, providing a cutting-edge analysis of the sensors along with the earlier methodologies and technologies involved in the current situation to facilitate the system [16,17,18].

This paper includes all the articles and review papers that are closely related to the sensors deployed, IoT, and cloud implementation in the healthcare domain. The analysis of the groundwork of the sensor implementation in the variable field along with the feasibility study relies on obtaining approval from organizing bodies such as FDE and CE. This also provides insights to hundreds of the papers included in the review along with the methodologies the fellow authors put together.

The process of a paper selection criterion is divided into three subsections: selection of the keywords, inclusion, and exclusion, and the final results that are obtained using these methods. They are explained below.

#### 1.3.1. Selection of Keywords

A thorough search was conducted across many famous databases for white papers such as PubMed, IEEE, science direct, etc. For those databases to search the papers, the keywords were selected such as wearables, IoT, healthcare, E-health telehealth, diabetes, real-time monitoring, movement tracking, fitness trackers, etc.

#### 1.3.2. Inclusion

Articles that were published after 2013 were considered for the study, and the rest were excluded. These shortlisted papers, when analyzed using the abstract as the center point and papers specifically mentioning the application of wearable technology, Internet of Things, and their relevancy to the research we were conducting, were considered for the review. This paper consists of a detailed review of the research articles, recent review paper, technical notes, etc., arranged in a systematic order related to the recent advancements in IoT, healthcare, and wearables.

#### 1.3.3. Exclusion

While searching for the research papers, the duplicate papers, the papers that are not relevant to the survey, the papers with irrelevant information, and those in languages other than English were excluded from this review. Papers were also excluded if they do not have any relation to wearable technology and provided already-existing information on the same domain. The case reports, case series, letter to the editors, commentaries, editorials, correspondence, short communications, etc. were not considered for the review.

#### 1.3.4. Result

**Figure 2:**
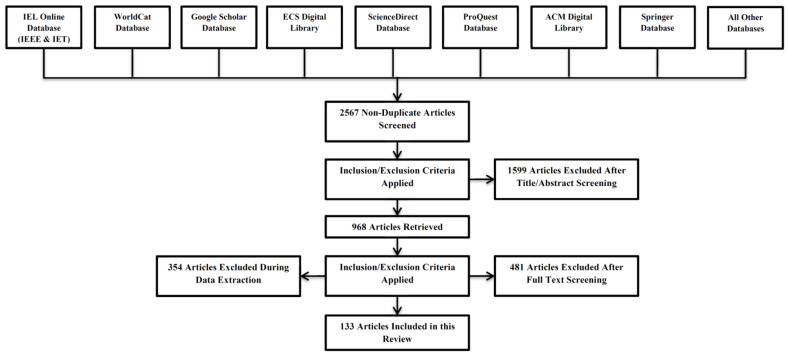
Prisma flow diagram for the selection process of the research articles used in this review.

After going through over 2567 papers in the initial stage using the abovementioned steps, we reduced the number of the papers to 968. After excluding the papers by reviewing the abstract and later full review and then analyzing the relevancy of the papers to topic of discussion, a total of 133 papers as shown in Figure 2 was selected for the detailed study for this review paper.

## 2. IoT-Assisted Wearable Sensor Systems for Healthcare Monitoring

In most systems, a simple network made up of wireless devices can be seen. Through this, a simple yet effective network to sense, record, and transmit data is created. A fair trend in wearable IoT devices in which existing and available sensors are used to detect, transmit, and analyze the data can be noticed. Over the years, several prototypes have been built with different sensors such as ECG, RFID, BP Sensor, and PIR Sensor [19,20,21,22,23]. Additionally, the field also uses microcontrollers such as Arduino, STM32 Microcontroller, ARM7, Intel Galileo, and Raspberry Pi, [22,24,25,26,27,28]. The communication protocols used were MQTT, BLE, GSM, ZigBee, LoRaWAN, and GPRS [27,29,30,31,32,33,34,35].

### 2.1. Architecture of IoT-Assisted Wearable Sensor Systems for Healthcare Monitoring

**Figure 3:**
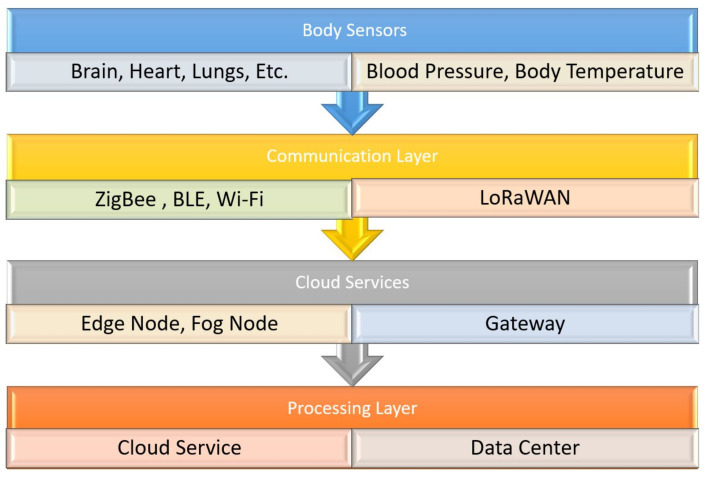
A brief overview of IoT-assisted wearable sensor systems for healthcare monitoring.

A host of different wearable sensors is used to create a WSN to monitor the patient remotely. The basic layout of an HMS is a sensor (or sensors) that is, in most cases, wearable. The sensor’s data are sent to the cloud via a communication protocol like Zigbee, Bluetooth, or Wi-Fi [36]. These data are then sent via a communication layer to the data center for further processing. The same data are visible in real time to the doctor, patient, and the patients’ caretakers to catch any emergency. This architecture can be seen in Figure 3, displayed as a flowchart.

### 2.2. Data Processing

**Figure 4:**
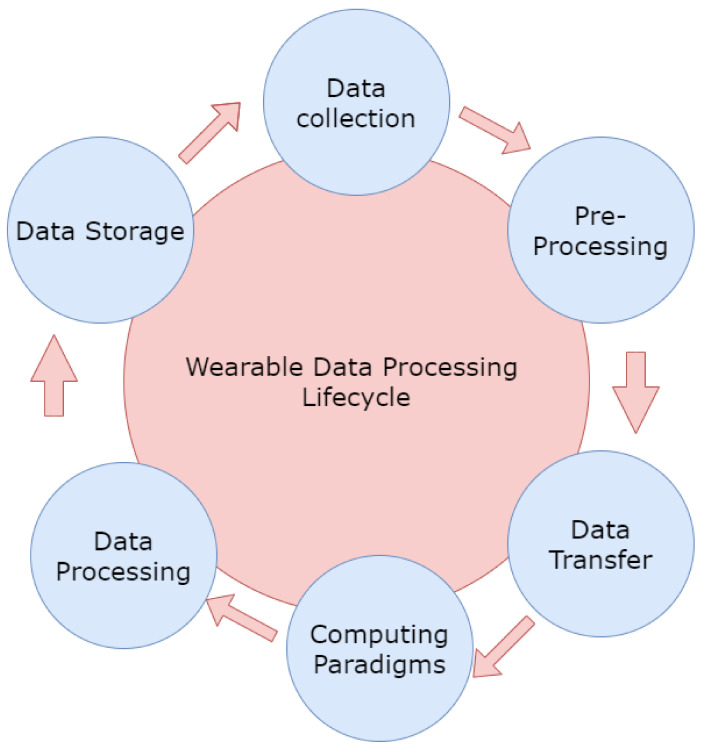
Data processing life cycle—IoT-assisted wearable sensor systems in healthcare.

For a wearable data cycle, the data are collected and then processed. The data are used for preprocessing where the outliers and the invalid data are removed. The data are then transferred to the computing paradigms, and we process the data using various machine learning techniques. It is then stored and collected via the cloud. This continuous cycle can be seen below in Figure 4.

#### 2.2.1. Data Transfer

A data transfer protocol is a standardized format for transmitting data between two devices [37]. The different types of data transfer protocols are:

FTP: File Transfer Protocol. Enables file transfer between remote systems.
UDP: User Datagram Protocol. Computer applications use this to send messages.
TCP/IP: As of now, it is seen as the most popular network globally. Most commonly used to this day for computer-to-computer communication.
HTTP: The Hyper Text Transfer Protocol is used for distributed and collaborative information systems. Tim Berners Lee developed it in 1989.
MQTT: Message Queue Telemetry Transport is used for machine-to-machine communication. It works on top of the TCP stack. It is the ideal messaging protocol for IoT [35].
LoRa: The LORAWAN Protocol is a long-range, low-power, low-bitrate communication solution for IoT. It works for battery-powered devices where energy consumption is a significant concern. It is long-range and low power [32].

#### 2.2.2. Computing Paradigms

In this subsection, different functional computing paradigms are defined:

Distributed Computing—Multiple computers work on the same problem. The problem is divided into different sections for different computers.
Parallel Computing—Here, different computer systems work simultaneously. The problem is broken down into smaller sections and are executed on different processors.
Cluster Computing—In this, multiple computers work together as a single machine to complete a task.
Grid Computing—A network of computers form a data grid to complete a task that might be difficult for one machine to do. Together, they can be seen as a virtual supercomputer.
Edge Computing—In edge computing, more processes are moved to the IoT device, edge server. This is performed to decrease long-distance communication between the client and the server.
Fog Computing—Fog computing is used to improve overall network efficiency and performance. It acts as a structure between the cloud and the data-producing devices.
Cloud Computing—Cloud computing means using a foreign server to host data. It is an on-demand service. Some well-known vendors of cloud services are Google Cloud, Azure, AWS, etc.

### 2.3. Communication Technologies

In this section, four different communication technologies popular in IoT networks are discussed. The four technologies discussed in detail are ZigBee, LoRaWAN, Wi-Fi, and Bluetooth. ZigBee, Wi-Fi, and Bluetooth are short-range technologies, while LoRaWAN is a long-range technology.

There are many more wireless communication protocols, but these four seem the most suitable for our purposes and needs. The technologies are compared in Table 2, at the end of the section.

#### 2.3.1. ZigBee

ZigBee is based on the IEEE 802.15.4 standard. It thrives in a resource-contained environment with power-limited devices. It forms a WPAN (wireless personal area network). It was initially conceived in 1998, though it was standardized in 2003. It uses the DSSS (direct sequence spread spectrum) technique.

ZigBee has a maximum range of 10–100 mts and has a meager power consumption. The network topologies ZigBee uses are ad hoc, star, and mesh. It has a data transfer rate of 250 Kbps and a channel capacity of 5 MHz. It is a very low-cost network.

The ZigBee has the following devices: a ZigBee coordinator (ZC), a ZigBee router (ZR), or a ZigBee end-device (ZED). The ZR is the trusted root of the network [38]. ZR is an intermediate router in a ZigBee network. The ZED has sensing capabilities. It communicates with the parent device. ZigBee is used increasingly in smart homes and home automation [41].

#### 2.3.2. LoRaWAN

LoRaWAN is an LPWAN (low-power wide-network technology) that has gained attraction in recent years for its need in IoT networks [32]. It is used to transfer a large amount of data over long distances. Because of its robustness and range, it works very well for WSN. It has a physical layer called LoRa (long-range), which Semtech creates. LoRa radio uses frequency shift keying (FSK) or chirp spread spectrum (CSS).

The data transfer rate of LoRaWAN ranges from 250 bps to 5.5 kbps. It uses bandwidths of 125, 250, or 500 kHz. The technology has two types of devices: a node and a gateway. A node sends and receives information from the gateway. A gateway is connected to thousands of nodes at a time. They use a star of star topology. The routing topologies suggested for LoRaWAN are tree topology and flooding approach [39].

#### 2.3.3. Wi-Fi

Wireless fidelity (Wi-Fi) is a wireless communication technology based on the IEEE802.11 standard. Wi-Fi uses the CSMA/CA protocol to access radio channels. A set of wireless stations and access points make up a BSS (basic service set) defined by BSSID (basic service set identifier). BSSID corresponds to the MAC Address.

Wi-Fi has a range of up to 10 m with very high-power consumption. It uses the point-to-hub network topology and has four different configurations: infrastructure, ad hoc, bridge, and repeater, and has a data transfer rate of 6.75 Gbps and a channel capacity of 160 MHz with a very high cost [36].

#### 2.3.4. Bluetooth

Bluetooth is based on the IEEE 802.15.1 standard. It works within a short-range and is used to exchange data between both fixed and mobile devices. It uses the master–slave model for communication. Bluetooth 5.2 has better power consumption, more robust security, a higher data rate, and an extended range than the previous versions. Bluetooth communicates via an authentication procedure called pairing.

Bluetooth uses three kinds of pairing mechanisms Legacy Pairing, SSP, and SC. Legacy Pairing uses a PIN code or a passkey to authenticate pairing. SSP (simple secure pairing) uses the ECDH (elliptic curve Diffie–Hellman) key establishment protocol [40]. SC (secure connections) upgrades the SSP with longer keys and better algorithms.

Bluetooth has a range from 10 to 100 m with medium-range power consumption. The network topology it uses is point to point or point to multipoint. The data transfer rate is 2.1 Mbps and has a channel capacity of 902 MHz.

### 2.4. Interoperability

Interoperability is essential for healthcare products to work seamlessly with each other. This can be achieved with device manufacturers following set standards or having standards for wireless communications such as ZigBee, Wi-Fi, etc. The manufacturers should also ensure that gateways are available for translating and transmitting data to different devices.

### 2.5. Privacy and Security

Sensor networks deal with two focal issues. The first is data oriented, and the second is context oriented. Data-oriented privacy is focused on securing the data integrity of collected and transmitted data from sensing systems [7]. It is straightforward to manipulate the data with wireless devices as it can quickly be performed by faking the data. Once the parameters are used in the WSN, the data can be edited accordingly. This would destroy the integrity of our data and be harmful to any future research in the plans.

Context-oriented privacy prevents the attacker from getting contextual information concerning sensor data collected and the location of data sources. To increase the privacy of our data, two-factor authentication can be used.

Security: WSN is vulnerable to attacks due to wireless communication and large-scale network. To prevent security attacks, we use cryptographic mechanisms to encrypt and decrypt data. RSA and Diffie–Hellman-based cryptography can be used in tiny sensor nodes [7]. A cyber-physical system, CPS, technology can also be used for HMS [42].

## 3. Applications—IoT-Assisted Wearable Sensors for Healthcare Monitoring

IoT-assisted wearables are widely being used these days. The friendliness of such devices has been a boom in the applications of their usage in all of the fields. With the healthcare field being no exception, the exploits of the IoT in healthcare are enormous. Various technologies are linked to existing technology that helps generate the data for monitoring and analysis. There are many applications of wearable sensors. For instance, a fitness tracker which is manufactured by various companies. The sole aim is to monitor the person’s pulse, movements, etc., by calculating the steps using the GPS and the accelerometer to figure out what type of activity the person is performing. Taking their weight, height, and age, the software can calculate the number of calories they have burnt, the number of altitudes they have been to, the number of stairs they have taken, average pulse rate, and much more.

**Figure 5:**
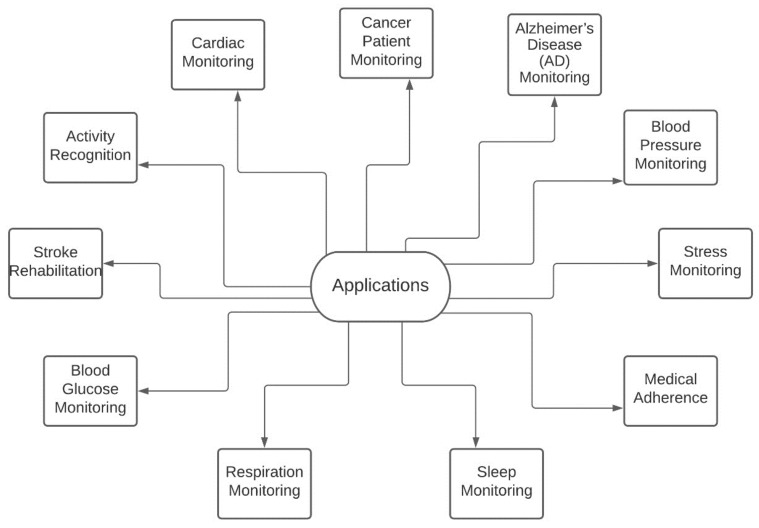
Application of IoT-assisted wearable sensor systems in healthcare.

There are various personalized services such trackers perform. Some have come up with calculating the SpO2 content in the blood to counter the current pandemic. Moreover, the readings themselves are pretty accurate. Due to this reason, some minor tweaks in the construction of the wearables can make them excellent equipment to capture the patients’ vitals, and due to Wi-Fi and other connectivity technology explained in the previous section, it can become cloud-based. In this study, the focus has been on the various papers and research articles. The applications of these IoT-enabled wearables are shown in Figure 5.

### 3.1. Activity Recognition

**Figure 6:**
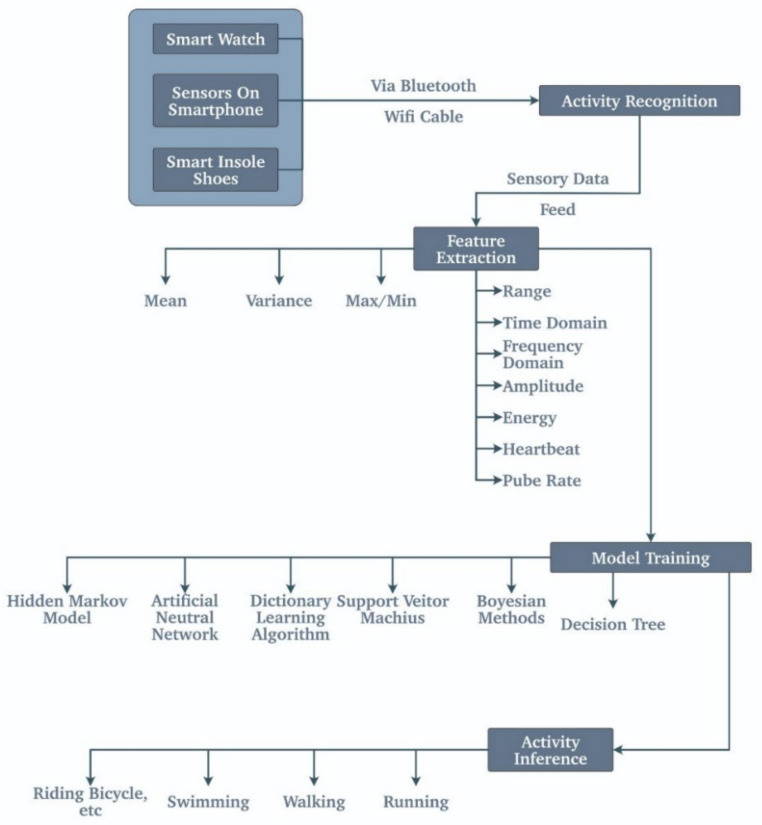
A brief overview of activity recognition methodology.

This is one of the widespread applications of healthcare wearables these days. Almost all fitness trackers do this type of recognition. Nowadays, fitness trackers are the prime wearables that are used to track the activity of the person. Most of them have a highly sensitive 3D accelerometer that enables the sensor to calculate the acceleration, and there is a lot of guess work going on in the background. Moreover, due to this calculated guess work, the wearable computes whether the user is walking, running, or sleeping. There is also a sensor that is inbuilt in such wearables to calculate the height above the sea level, and it is due to this that they can track the number of flights steps one takes. Figure 6 briefly covers the activity recognition methodology used in these wearables.

The tracker uses a mobile application to run and synchronize its values. Usually, these applications often take the person’s height, weight, age, gender, and other personal details while setting the application on the smartphone for the first time. These data are crucial in calculating the steps and differentiating the activities they are doing. These companies also use their data to train the application to predict the movements, activities, height, weight, BMI, etc.

### 3.2. Stroke Rehabilitation

Heart diseases are very hard to counter if there is no proper management system to manage the patients. Uttara Gogate et al. have made use of the WSN for cardiac patients. According to various studies, heart diseases are prevalent in older adults [43]. Their health can take a turn at any point. This includes urgent and critical situations. The heart patients require continuous monitoring so this system can be used as a real-time monitoring system using the WSN technology. This WSN includes various medical-grade sensors and equipment that can monitor the heartbeat, pulse rate, body temperature, and blood pressure, and critical patient’s real-time ECG is maintained so that the patient is monitored continuously.

The above system mentioned is also used to take good care and monitor the patient’s postcardiac arrest situation. Patients are very vulnerable in postcardiac arrest situations, and the system discussed earlier would be quite helpful in this scenario.

**Figure 7:**
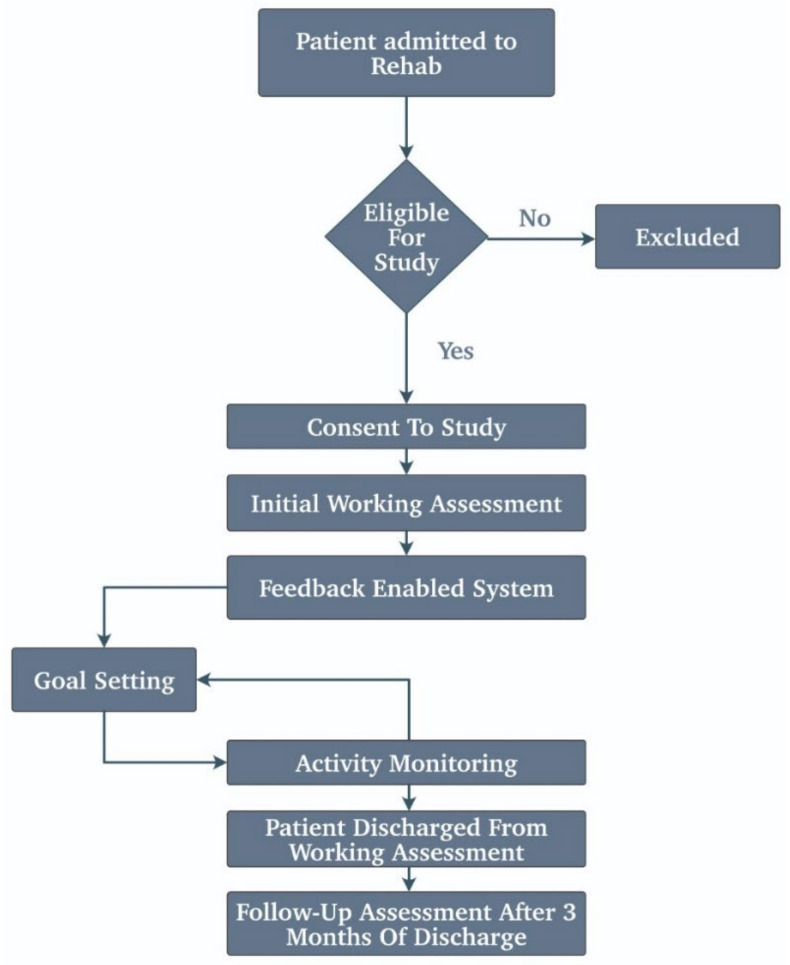
The algorithm used for stroke rehabilitation (recovery of motor controls).

Apart from cardiac arrest, other strokes hamper the motor controls of the body to such an extent that the brain nearly forgets to send the electrical impulses to control the hand movement or the leg movements. There has been an evolving technology to counter such techniques where the IoT plays a considerable part. There are special units named the IMUs that are solely used to counter such a measure. The authors have a detailed study on this specific topic. It uses the IMU to correct the movements of the body. These IMU create a body area network that makes the readings entirely accurate [44]. Figure 7 illustrates the working of the algorithm in rehabilitation to recover the motor control.

### 3.3. Blood Glucose Monitoring

**Figure 8:**
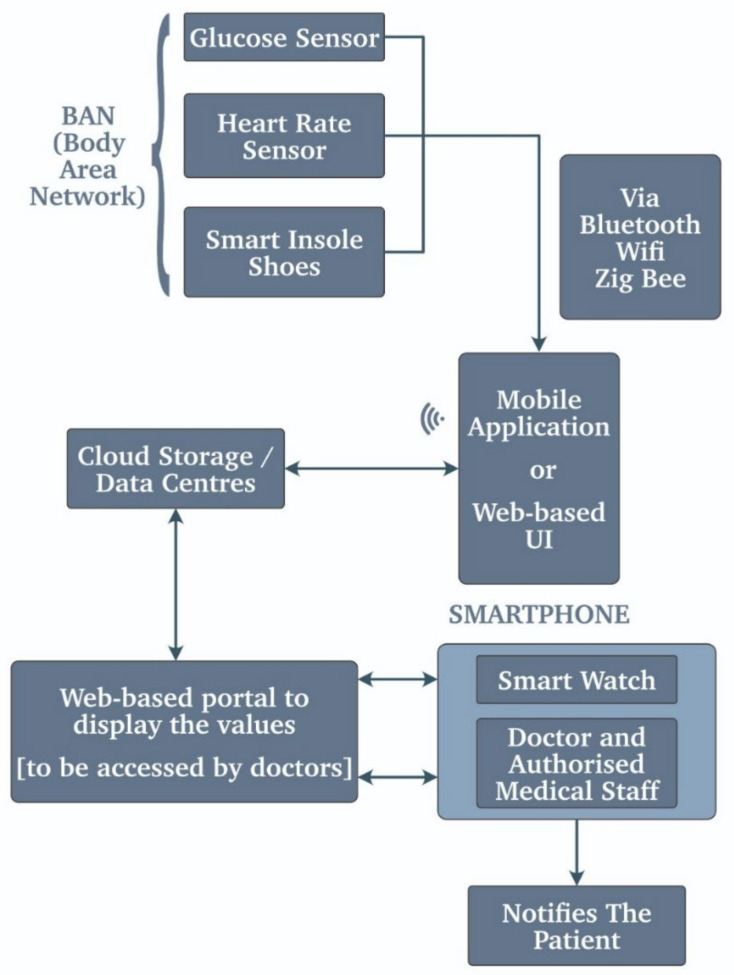
Blood glucose monitoring methodology.

For diabetic patients, the use of such IoT devices has made considerable progress that is commendable, and due to this progress, the accuracy of glucose monitoring in the blood has increased exponentially. The use of such vitals while monitoring a critical diabetic patient can be lifesaving [45]. IoMT stands for Internet of Medical Things, and as the name suggests, it is the IOT specially designed for the medical field [45]. The IoMT consists of the heart rate sensor, blood glucose monitoring, endoscopic capsules, etc. and this interconnected network of sensors that communicates using IoT forms the IoMT diabetic-based WBSN monitoring system. The proposed system concluded that it was robust, flexible, and cost-efficient and used medical-grade sensors and technology, making it more of a reliable option than the other IoT-based similar system. There are various other cases where we have considered the use case of glucose monitoring in [6]. They developed a healthcare monitoring system that focuses on monitoring the necessary whole-body vitals, including blood glucose. Figure 8 also illustrates a similar kind of system that uses a body area network, a mobile application, or a web-based UI and sends the data to the smart watch and the authorized doctors, who in turn notify the patient about the situation.

Moreover, it sends it via a network to the cloud via an Esp8266 WIFI module serially. CPS continuously monitors the patient’s health parameters such as blood glucose (BG) level, blood pressure (BP) level, body temperature (BT) level, and heartbeat (HB) rate. They describe a CPS framework [42]. The fundamental aspect of data storage should be single and reliable—organized cloud data storage (OCDA). They use an EMG sensor to measure the changes and disbalance between the neurons and muscles. All the data are shown on the LCD [46]. A GSR sensor is used to check the sweat gland activity and an INA219 sensor to check the glucose level. Electroencephalography uses an EEG sensor to trace the electrical activity of the brain. Finally, they used an MPXV5050GP sensor and LM35 integrated circuit for blood pressure and body temperature, respectively.

### 3.4. Cardiac Monitoring

Cardiac patients need a dependable health monitoring system that oversees all the vitals and sends alerts to the concerned authorities whenever there is an emergency. Uttara Gogate et al. have made use of the WSN for cardiac patients [43]. This includes urgent and critical situations. The heart patients require continuous monitoring so that this system is used as a real-time monitoring system using the WSN system. This WSN includes various medical-grade sensors and equipment that can monitor the heartbeat, pulse rate, body temperature, and blood pressure, and for the critical patients, a real-time ECG is maintained so that the patient is monitored continuously. The body parameters are recorded successfully and are shown serially, and along with it, the data are sent to the cloud and can be accessed by the doctors. If any abnormal reading occurs, an alert message is sent to the caretakers. Uma Arun et al. have used the existing National Instruments (NI) LabVIEW hardware known as the myRIO 1900, similar to NI myDAQ. This NI myRIO is used to connect to the ECG sensor, and the NI LabVIEW can be used to use the myRIO to obtain the sensor output. This system is solely made to benefit people with cardiac diseases long facing them [47]. The vernier EKG sensor senses the potential differences and sends the values to the LabVIEW software. The software understands the reading and graphically shows them on the screen. The paper also shows the detailed diagram for the LabVIEW that can make such a system to obtain the output properly.

The system depends on the Internet of Things (IoT) technologies and is most suitable for patients with chronic cardiac disease [30]. The system’s primary functions are the following capabilities:

(1) obtaining body sensors’ data;
(2) analyzing health status on a smartphone;
(3) transferring bodily information to smartphones through a body sensor network and a wireless body area network using the ZigBee and Bluetooth technology.

Some systems suggested Arduino Uno, LM35 body temperature sensor, DHT11 humidity and temperature sensor, AD8232 ECG sensor and ADXL335 body position sensor, MAX30105 heart rate monitor sensor, Bluetooth 4 BLE module, 16 × 2 LCD, R pi, and WIFI/4 g LTE. The algorithm is proposed in 10 steps with a proper explanation [11]. The slave circuit consists of all the sensors connected to the Arduino Uno and a push-button display. The program of the slave circuit is also given, which is written on Arduino IDE using embedded C. The master circuit includes the R-pi, WIFI module, and Bluetooth module. The master code is written in python.

### 3.5. Respiration Monitoring

There are several ways we can monitor the respiratory system in the human body. Some authors used specialized sensors that monitor breathing movements. Using a bioimpedance sensor can come in handy [30]. This sensor is multifunctional as it sends a small amount in the skin and then calculates the respiration movement and the heart rate at a rough scale. The system proposed also uses a specialized respiratory sensor to send the analog values to the MCU [48]. The sensor is attached to the abdominal area of the patient. This proves that the wearables cannot measure the exact respiration until they attach the sensor to the abdomen, and that too should be the medical-grade sensor.

### 3.6. Sleep Monitoring

This sleep monitoring application helps the person correct their sleep cycle and keeps a healthy life cycle going. Various sensors are used in this section. Some wearables usually monitor the heart rate, pulse rate, SpO2 levels, and breathing patterns and make a calculated guess about sleep quality by considering the parameters.

These wearables are primarily multifunctional and also use the GPS, three-axis accelerometer, and altimeter to determine whether the person is asleep or not. Moreover, due to these features, fitness bands are now an integral part of people’s lives.

**Figure 9:**
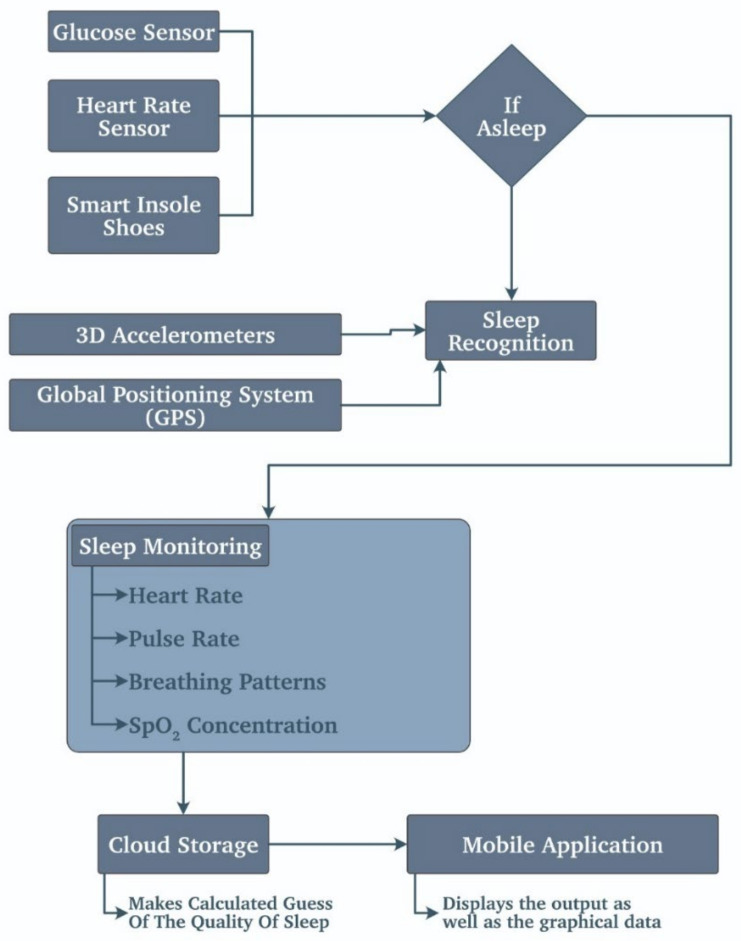
Sleep monitoring in real-time using WSN.

An IoT gateway as an intermediate hub between the physical layer (sensor nodes) and the server has been developed for data collection and synchronization to facilitate efficient end-to-end communication between user and medic in real-time [49]. The results indicate the monitoring of users while asleep. It shows the user is experiencing a sleeping time in standard environmental conditions (see ambient parameters). A couple of noise spikes were observed but instantly disappeared in the results, but this occurs only a few times and cannot cause serious interference. Heart rate, skin temperature, breathing rate, and level of sleep are depicted and analyzed. Figure 9 describes the sleep monitoring system mentioned above using WSN.

### 3.7. Blood Pressure Monitoring

Blood pressure is so widespread nowadays that almost every fifth person in the world suffers from a mild BP problem. Moreover, the healthcare department also does not take it lightly. The high shoot in BP are signs of various chains of action in the body. This high rise in BP is a type of stimulus of a patient’s physical and mental wellness. When one is depressed, the BP of the person also changes accordingly; therefore, to monitor anything in the human body, there is a need to monitor the BP for sure.

To calculate the BP, there are various ways to measure it. Most of the doctors use a sphygmomanometer to calculate the BP. Moreover, for the wearables, they use the heart rate monitoring system to work this out. Using the pulse wave analysis of the reading by the pulse oximeter, the wearables use the specialized algorithm to obtain the estimated BP by considering all the parameters such as the age weight the previous data collected and estimate the approximate BP of the user. As we know that the heart diseases are prevalent in older people, their health can take a turn at any time. This includes urgent and critical situations [43]. Heart patients require a continuous monitoring system, and a possible solution is using the WSN system. The WSN system can monitor the heartbeat, pulse rate, body temperature, and blood pressure, and for the critical patients using its medical-grade sensors, a real-time ECG is maintained so that the patient is monitored continuously. They have tried to develop a data monitoring system for pregnant ladies. Their model would analyze the blood pressure, temperature, heartbeat, and dental movements [50]. The perfect tissue differentiation would be conducted using the ultrasonic sensor (medical grade). Using this ultrasound, the clear picture of the soft tissue can be visualized and thus monitored in real-time. The system has been proposed that used the R-Pi as the central processor, and they intend to use the temperature sensor LM35 that measures body temp, the BP sensor to record the blood pressure. The heartbeat sensor and ECG sensor obtain the vital body signs from the patient [51]. They have interfaces with all the sensors to the R-PI module, and the sensor data are sent to the R-Pi module for further analysis. The data are thus saved locally with the R-pi and routed to the cloud for further analysis. Using the tools such as MATLAB and LabVIEW, the visualization of the data is taken place. Moreover, the alert message is sent to the prison responsible via the GSM module if the readings are not expected.

**Figure 10:**
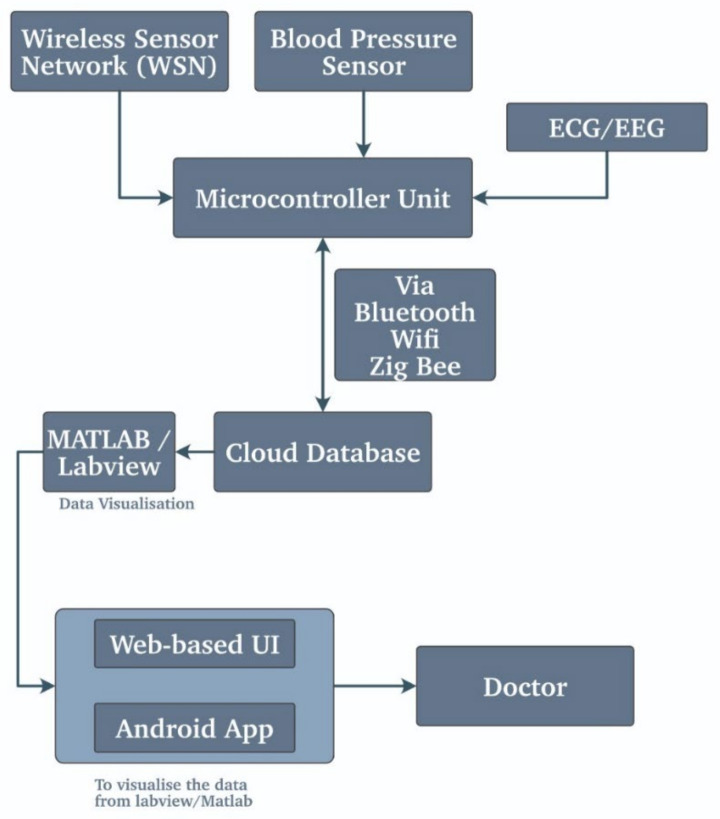
Blood pressure monitoring system.

This use case of the wearable devices of calculating the blood pressure is often used in almost all the areas, whether it is a calculation of mental health or any other test. In this field, the need to use the BP values has proven way more advantageous. Whether it may be an intelligent health monitoring system [49] or the portable system [46], it is necessary to calculate the BP. Figure 10 describes a similar point of view of the blood pressure monitoring system explained earlier.

### 3.8. Stress Monitoring

**Figure 11:**
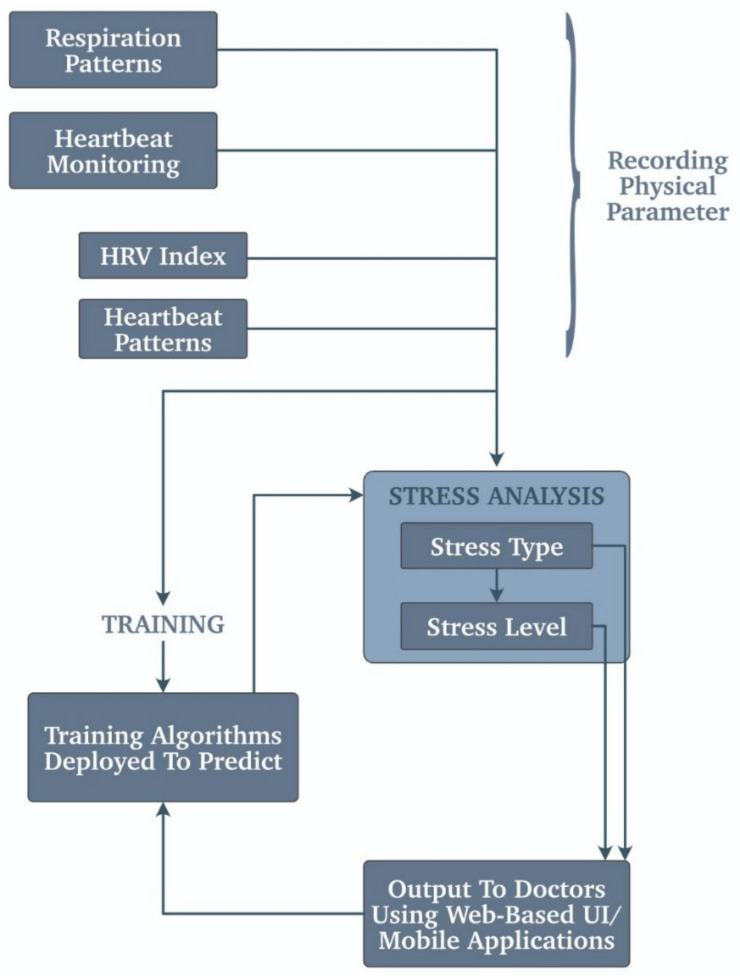
Stress monitoring system.

Stress monitoring is performed by taking all the body’s vitals and then comparing them with the readings at rest. Whenever the BP or insulin levels drop in diabetic patients or there is sudden uneasiness in breathing, these are the unmistakable signs that point towards high stress, and the wearables that a person is wearing monitor and remind the user to keep it slow. The alerts can be a reminder to drink water or an everyday gentle reminder that gives the user a short relief that they are not alone or anything that boosts the patient’s morale. The sweat glands also have a part to play in stress monitoring [52]. Further, not only sweat but also minor changes in body fluids also specify that the person is under stress. Typically using TSST (Trier social stress test), the actual stress can be found out. This technique cannot be used with the wearables but is dependable. Figure 11 shows a similar scenario of a stress monitoring system using the respiration patterns, heartbeat monitoring, and HRV index. HRV stands for heart rate variability that is the fluctuation in the heartbeat pattern over a while.

### 3.9. Medical Adherence

Medical adherence is also a critical application in healthcare. It is vain to treat the patient if they cannot follow the prescribed medicine or the advice that the doctor has given him/her. Such patients later cannot be helped even if they wanted to. In such cases, this application comes in handy. Some systems and models are proposed for this, such as e-health [9]. The e-health systems are capable of vending the prescribed medicines after examining the patients.

Furthermore, the backbone of these e-health systems is the IoT, and a doctor or any other trained personnel can be appointed to supervise the system. This saves the doctor’s time in seeing the less critical patients, which such a system can handle, and now the doctor can pay his/her full attention to the critical patients where his/her skills have a part to play. The remote diagnosis of the patients is also enabled because of this system. There is a system shown as EHMS [53], i.e., the e-healthcare monitoring system. The EHMS system is solely designed to check and manage the health monitoring system over the internet. All the patients’ vitals, such as the ECG, the heart rate, the SpO2 concentration, etc., are analyzed and monitored.

It deals with a low-cost health-monitoring system focused on rural areas. It discussed various remote healthcare systems that collect data using wireless sensors, queries, and communication using telephone lines. The proposed method consists of hardware that can collect data such as images of body parts at a proposed sampling rate depending on the patient and has a VC facility. It also can work offline. It uses DAQ (advantage: single channel for all sensors). Local storage; upload when a good connection is available. It gives secure login to users. It involves a home screen for login. The website works with a unique reference ID for every user. The doctor interface has a list of patients and their sensor data [54].

**Figure 12:**
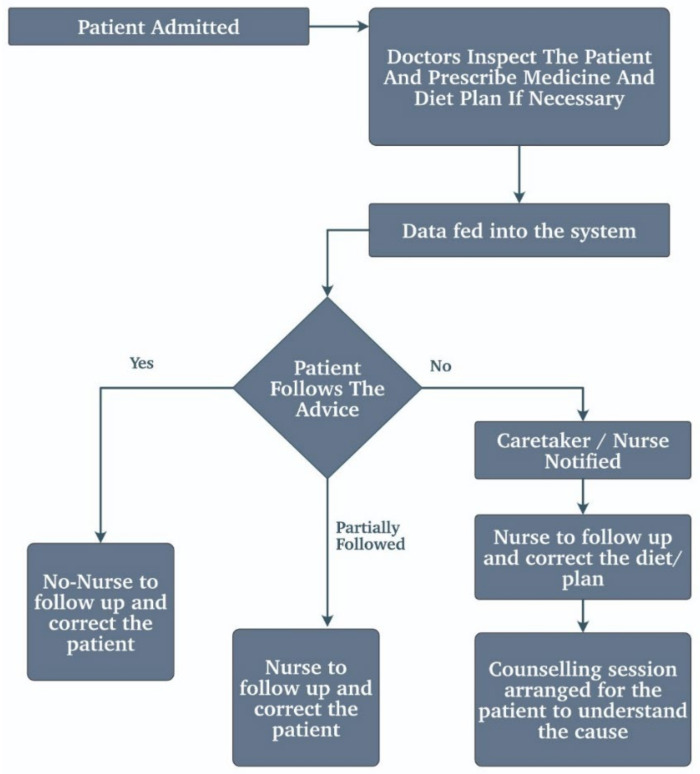
Medical adherence methodology.

Some models are doing the same thing as the medical adherence using the above e-health system to get alerts to elderly people. One of the models is explained in Figure 12. This model can also be applied to elderly people as well as to anyone who is not good at following doctors’ advice.

### 3.10. Alzheimer’s Disease (AD) Monitoring

Alzheimer’s disease monitoring has posed many problems and needs to be handled with the utmost care. Patients with Alzheimer’s disease cannot be diagnosed when they are on their own. Furthermore, even for the family, it is difficult to observe the disease. They can only find out about the disease when the patient acts weird and loses consciousness when doing simple tasks. The authors believe that the IoT can play a massive part in countering or easing the patient’s quality of life suffering from AD. There are three primary symptoms of AD that are particularly dangerous:

1. Severe memory loss
2. Wandering
3. Dementia [55]

To counter this, the use of wearables connected to the BAN with the help of communication protocols such as the MQTT, Zigbee, etc. The sensors can alert caretakers about the patient’s location when they wander off in the city, making finding them easy for them. Even the patients can be trained to use the IoT framework to obtain notes or remember things such as their name, the doctor taking care of them, or their address. Moreover, further advancing the technology using the various servers and databases to collect the vitals from the patients can make it easy even for the doctors to monitor the patient even if they are not around.

### 3.11. Cancer Patient Monitoring

Cancer is a disease that does not have a proper treatment without any side effects. The tumor has to be removed using chemotherapy alone, which causes the patient to be weak and unhealthy. It is essential to take care of the patient in such a condition as it is vulnerable to almost anything. In this paper, the author has made an effort to develop an IoT-based framework and a layered architecture. They have defined five layers for this system [56]:

1. Service layer
2. Datacenter layer
3. Cancer care layer
4. Hospital layer
5. Security management layer.

All the layers are self-defined by their names.

This system uses medical-grade sensors that can detect the tumor cells that are present in the patient. There would be cloud support and analytical skills to help the doctors decide on specific urgent situations. The sensors would be connected to WSN technology and other intelligent devices and facilitate sending the data across the globe. They have compared the proposed system to the existing one and found out the system was sound in almost every way.

## 4. Wearable Sensors

All IoT-based healthcare systems include a sensor layer, which collects data from the user/patients by measuring their vitals and other necessary signals and converts it to data that can be processed and monitored. This layer may include various sensors that can detect activity and monitor vitals such as pulse, oxygen level, temperature, glucose, and other specific signals indicating an abnormality. These sensors are primarily wearable, given that the users must be able to continue with their daily activities and go on with their lives uninterrupted. A lot of these systems use BAN or WSN for networking. Moreover, the data stored in the cloud are then preprocessed, trained, and used to predict ailments using various algorithms [12] and AI [57]. These sensors can be categorized into different types discussed in the subsections. Table 3 is a compilation of various wearable sensors used in IoT systems in healthcare, with their application in various research works.

### 4.1. Activity Detection Sensors

Activity detection sensors are sensors that detect and monitor the movements of a person. It can be specific to a part of the human body. Much information can be collected and used to monitor a person’s health based on their movement. Older people are more prone to physical damage, and they must be monitored for unnecessary movements that might indicate danger. The most common sensor used is an accelerometer. Accelerometers are devices that measure the acceleration of the body it is attached to. Using the sensor data, we can detect the patient’s body movement and set thresholds that can indicate possible dangerous movements such as falling or slipping [26]. The paper [22] builds on the healthcare system that monitors movement and other data and transmits it to the cloud for processing. The device in [23] describes a system with a PIR sensor to detect movement and other data as part of a healthcare monitoring system. The system in [73] uses an ADXL345 for observing the movement of patients and records it.

### 4.2. Respiration Sensors

Respiratory sensors are an essential peripheral in IoT-based healthcare systems. They monitor the gases inhaled and exhaled and the breathing rate of the patients. It includes a pulse oximeter, airflow sensors, and oxygen sensors. A pulse oximeter is a clamp-on device that is a noninvasive method to calculate oxygen saturation in the blood. It passes light beams through the blood in the finger or earlobe/toe and measures light absorption changes. Nasal/mouth airflow sensors are devices that monitor the breathing rate of users. It is a flexible pipe with two prongs that go into the nostrils and sit on the ears. The paper [53] describes an e-healthcare monitoring system that checks and manages health through the internet using the SPO2 and other heart monitoring sensors. References [54,66] use a pulse oximeter sensor to sense the blood oxygen saturation and to find the pulse of the user [3,19,45]. Reference [33] explains a model that uses R-pi and interfaces an oximeter and airflow sensors to monitor the patient’s breathing [51]. The device on [46] uses a MAX30100 integrated sensor to check the oxygen level in percentage and specify the normalcy range between 94 and 100. The system in [67] uses a MAX30102 sensor to detect heartbeats and calculate capillary oxygen saturation using reflected mode. The paper [68] uses a pulse oxygen sensor to calculate the amount of oxygen dissolved in the blood by detecting hemoglobin and deoxyhemoglobin.

### 4.3. Heartbeat Monitoring Sensors

Heartbeat monitoring sensors work using the principle of reflection of light through a vascular region of the body. Monitoring a person’s heartbeat can help anticipate many healthcare issues that are normally not easy to detect or not recognizable symptoms. ECG sensors [53,61] measure the heart’s electrical activity and represent it as a graph. It is used majorly in all healthcare systems to detect heart conditions and helps identify chest pains and other common symptoms. The research in [80] deals with anomaly detection in the ECG readings taken, using filters, and calculating the energy variances. The papers [81,82] discuss devices that contain a sensor to monitor the heart and other vitals as part of the automatic monitoring system [3,24,28,29,62,65,67,68,73,79,83]. The system in [43] details a wireless sensor network (WSN) [56,61] for cardiac patients to have continuous real-time monitoring that would be uploaded onto the cloud for analysis. Research in [47] describes a cardiac monitoring system that uses NI myRIO-1900 to transmit the data to NI LabVIEW, where it can graph [31]. The research [84] compares single-lead and multiple = 0 lead ECG recording devices and presents the results. Paper [20] discusses tracking the patient’s location and their heartbeat using a GPS module. The research in [83] outlines the effects of using wet and dry CVDs as ECG electrodes and their advantages.

### 4.4. Blood Pressure Sensors

The blood pressure sensor is designed to measure the blood pressure of humans through a noninvasive method. Monitoring blood pressure can help regulate the health of adults and anticipate health issues in the future. Data from the sensor can be correlated with other sensor data to discover any abnormalities in the patients [68,85]. The research on [86] describes a health monitoring system for sports athletes by continuously monitoring their vitals and transferring them to the cloud [5,22,24,29,48,53,77]. The paper [42] proposes a CPS framework, where the data are sent to various cloud storage systems. The device in [35] collects ECG and PPG data to estimate the blood pressure and transmit them to the cloud wirelessly through Bluetooth. The system described on [46] uses an MPXV5050GP sensor to calculate the blood pressure and interface it with an Arduino, which acts as a slave to R-Pi.

### 4.5. Blood Glucose Monitoring Sensors

Glucose sensors are designed to measure the glucose level in a patient’s blood and regulate diabetes. The sensor comes either in strips or as a strap-on sensor that can continuously monitor the glucose level in the body. A majority of the population benefits from using these sensors and monitoring their glucose level themselves. The research on [6] describes a noninvasive method to monitor the glucose level in the body using the ANS216 sensor. The system described on [46] uses an INA219 sensor to check the glucose level interfaced to R-Pi via Arduino. The paper [53] describes collecting blood glucose levels and processes the data using ML algorithms.

### 4.6. Temperature Sensor

Temperature sensors are ubiquitous sensors that can be used in combination with other sensor data to anticipate various health issues [22,61,63]. Temperature is the primary and direct indicator that there is something wrong with the patient’s body. The research in [70] describes a secured intelligent healthcare monitoring system that involves an array of sensors and is displayed for the user [24,65]. The paper [60] describes an intelligent system to monitor the environment along with body temperature data [19] and set off an alarm in case of crossing the threshold. The paper [62] proposes a system that works on body area network (BAN) with many sensors integrated into an MCU. The research in [5] describes a system where the data are collected and organized using RFID, and the data are transferred using the GSM module to the cloud [3].

## 5. Recent Advancements and Issues with HMS

### 5.1. Devices and Systems

The use of medical devices in hospitals and healthcare has been growing for the past decade, and it has made health monitoring easier for people around the world. Some of the common devices and systems that have been instrumented around us are listed below:

1. Wearable fitness trackers—Recently, the market for wearable fitness trackers has bloomed. People no longer depend on regular checkups. Rather, they prefer using trackers to record their vital signs and track their workouts and progress. Some common trackers that are available in the market right now are Fitbit and GymWatch.
2. Smartwatches—Smartwatches were initially meant to show time and connect to the phone and make it easily accessible. However, recently, they have been equipped with sensors and other systems that can monitor various aspects of the user and relay the information to their phone. Apple’s watch has recently been focusing on monitoring heart rhythms and informing people who experience atrial fibrillation. They have also released a “Movement Disorder API” to gather information on Parkinson’s disease.
3. Smart contact lenses—Contact lenses were developed to help people with their eyesight without wearing spectacles. Smart contact lenses help with monitoring the patient’s eye condition and collect data on changes in eye dimensions. These have been CE- and FDA-approved and are for sale in various countries.
4. Biosensor Patch—VitalPatch Biosensor has had issues with a EUA to be used in hospitals to monitor the changes in ECG who are being treated for COVID-19. This system helps monitor the heart rate of the patient without putting the medical professionals.
5. Blood pressure monitoring device: The first cuffless blood pressure monitoring system has recently been approved by FDA. Biobeat is a system with a patch and a watch that monitors blood pressure, oxygen rate, and heart rate. It has made self-care easy and intuitive for the elderly as well as for the long-term care of patients.

These are a few of the commonly found systems that have been developed in healthcare recently, and the comprehensive assessment of various technologies compiled in this paper hopefully helps further the cause.

### 5.2. FDA and CE Approval

FDA stands for Food and Drug Administration. FDA approves the medical equipment used in the hospitals, clinics, etc., in the USA. CE approval is needed if the items are commercialized in the European Union, which includes 32 different nations.

These two approvers are considered to be one of the best. It is difficult to be approved by them. If FDA approval for a certain medical product is granted, then it is assumed that it is safe to use in any part of the world. However, local authorities still check the device or a drug if needed. If a drug has FDA approval, the local government bodies are most likely to go easy on those drugs and take relatively less time as the FDA or CE approvals are performed on a thorough examination of the drug or device on different test subjects to approve the drug in the country.

CE, on the other hand, is a marking that states, a certain medical device follows the general safety and performance requirements (GPRS). The general safety and performance requirements are approved by the European Union and are granted permission to bring the product to the market. The CE mark is given to various products. It may be a medical device, a drug, or any electronic equipment. The CE mark guarantees that the product meets the standards of performance, safety, quality, etc., of the product type. The CE also grants permission for the product to be sold in the markets across all 32 countries in the European Union. All the maintenance, regulatory measures, security, etc., are the responsibility of the manufacturer of the product. The CE marks are taken away from any product if the manufacturer of the same product is changed. It is necessary to renew the certification every three years; otherwise, the product will be discontinued from the market [87].

#### FDA and CE Approvals for Wearables

Variables such as smartwatches, etc., were earlier considered to be types of clothing and were a standalone piece of technology. However, nowadays, there are smartwatches that can do numerous functions such as collecting body vitals activity recognition, serving as a multimedia device, and the native feature of showing time and serving as a digital stopwatch.

Technically speaking, if any device monitors health and body vitals, they are considered medical devices. In 2011, the first wearable technology by iRhythm, known as Zio Patch, was FDA approved. It was capable of monitoring a heartbeat for 14 days straight and was an alleged medical device back in the day.

In 2018, Apple proposed its smartwatch to the world, which had received FDA approval. The smartwatch contained a sensor that was able to accurately measure the ECG of a person and monitor the heart rate. Apple also claimed that the sensor used in the smartwatch was a medical-grade sensor and thus was approved by the FDA in the United States. This was the actual start of the FDA recognizing smartwatches as a device that enables the user to control their health. The FDA and CE are giving their special attention to “software as a medical device”. They also have a dedicated portal on their official website stating the guidelines for software’s quality of service.

## 6. Open Problem and Future Opportunities

### 6.1. Open Problems

**Figure 13:**
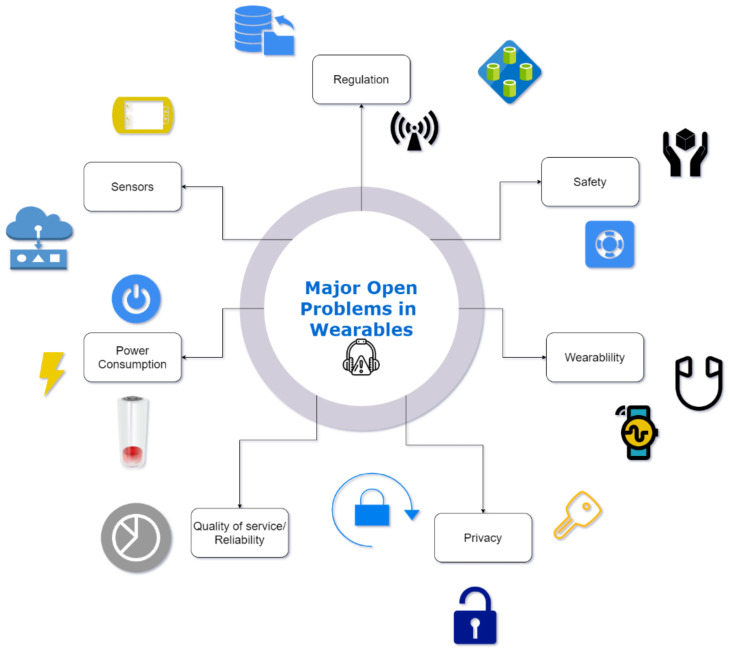
Open problems—IoT-assisted wearable sensor systems in healthcare.

IoT is a booming market in the technological sector, and such a boom in usage and potential usage makes many security issues. Several problems in cloud databases can hamper the IoT experience, leading to instability and the loss of trust for the users of the IoT. This phenomenon also applies to wearables as well. The wearables contain sensors responsible for collecting the data from the patient and sending the data via Bluetooth or Zigbee to the host devices. Many problems need to be addressed. The most significant problems are mentioned in Figure 13.

#### 6.1.1. Data Resolution

The data resolution problem is related to sensors. Most of the sensors used in the wearable sector need to be tiny to fit in a small-form factor. No one wants a substantial smartwatch clinging to their wrists that is not compact, sturdy, and sleek at the same time. However, there are some problems in selecting smaller sensors. The main and the most important one is data resolution. Due to the small-form factor of the sensor, the reading is very inconsistent; moreover, the range of the sensor is also limited, and sometimes some critical parameters have to be theoretically calculated in the MCU. This reduces the sensor’s accuracy and does not give the readings as compared to the medical-grade massive sensors.

For example, consider a simple heartbeat sensor. In hospitals, to monitor the heartbeat, there is a specialized sensor known as the ECG or EKG. This involves using a bunch of sensors that are attached to the abdominal part of the person’s body to obtain an accurate reading with all the due details, and the doctors use such detailed information to arrive at a conclusion, but in the wearables, there is a pulse rate sensor that is used to obtain a reading which is not as correct and detailed as the medical grade.

#### 6.1.2. Power Consumption

The wearables are used extensively, which creates a problem of powering the device. Since we know the law of miniaturization, the smaller the machine, the less power is consumed, but the more significant is the risk of damage due to excessive power. In the wearable world, the need for getting a dependable power source is necessary. Whenever there are many sensors to be used simultaneously, the need for power increases, thus making the situation worse, and the main problem is fitting all the sensors and a sufficient battery that lasts at least a day or two.

Today’s power issue is mainly solved using lithium-ion polymer batteries and a micro-PCB protection circuit to charge the device faster. However, when operating in a critical environment, power consumption should be handled with utmost care. We do not want the patients’ readings to stop for any reason abruptly. Based on the application of the wearables, the selection of battery should be made. Moreover, based on the battery, the type of protection circuit and charging circuitry should be deployed considering the size.

#### 6.1.3. Privacy

We know IoT has a massive threat to privacy. Earlier, the world had seen many privacy issues related to healthcare IoT. Various attacks on the network stopped the whole service for much time, money was lost, etc. However, when considered in healthcare, this problem has grown even more significant compared to commercial IoT. The data from this healthcare can be misused, which makes it very dangerous. This can be performed just by taking several sensor data by a smartwatch. The application can detect our sleep cycle, eating habits, the timetable that we follow, and much more. Thus, maintaining the privacy of the user data and the identity of the person is necessary.

#### 6.1.4. Wearability

The word ‘wearability’ is self-explanatory as it is. Wearability is the main factor of comfort. A wearable should be comfortable enough that the person wearing the thing should be able to use it throughout the day without any hindrance. The wearability of the wearable is mainly dependent on several factors:

1. The weight: the device should be lightweight.
2. The design should be ergonomic: the device’s design should be such that it matches the curves of the human body. It should not be sticking out from the body.
3. Water resistant: the device should preferably be water- and dust-resistant, as the device is meant to be worn on the body for at least a couple of days and can be used while traveling; therefore, there is a chance of water being spilled on it, and water resistance counters such minor hindrances.
4. The device should be made of a skin-friendly substance. It should not cause any rash of any kind to the person who is wearing the device.
5. The device should be soft, flexible, and durable at the same time.

#### 6.1.5. Safety

The sensors that are used in this section should be safe to use as well. The safety of the users takes the priority. The sensors and the device should be safe to be worn. There should not be any side effects of wearing it. Not only should it be safe to wear, but it should not be harmful to the body in the long term.

The wearable should be designed to affect the safety of the person wearing it and its people. However, these are not considered cheap and nonregulated devices but are still in the market, and ordinary people buy them because they are cheap. When used, these defective devices can be harmful to the person using them in all the ways and the people around the person wearing them.

#### 6.1.6. Regulation

As we know, IoT is new to the field of healthcare. Various companies are researching this field to provide the full support of wearable technology to monitor people. Moreover, the field is a niche, and because of it, there is no central authority to decide the regulations related to the devices that come out. Moreover, because there are no regulatory measures, many devices are not worth being used in healthcare centers that still come into the market and are available at a lower price than good and dependable ones.

Moreover, these devices are categorized as plain-old and not as healthcare devices in terms of wearables, meaning there is an obvious error here. The standards that a healthcare device should pass are not being used on the wearables for remote healthcare monitoring.

#### 6.1.7. Sensors

The sensors are the only part of the wearable devices, but everything revolves around the sensors being used. The sensors that are used in wearables should have the following characteristics:

a They should be small.
b They should consume minor power.
c It should not be very noisy.
d They should be easy-use compatible.
e Fairly accurate.

With the sensors being the soul and heart of any healthcare monitoring system, they should be accurate. However, the problem with wearable sensors is that they need to be accurate and small at the same time. The medical-grade sensors are big and very hard to carry around and require specialized equipment and trained personnel, i.e., doctors, to analyze the output of the readings. Like for the heartbeat sensor used in hospitals, there is a graph generated from the machine, known as an ECG graph, and after analyzing the graph, the doctor needs to figure out what is wrong.

However, in the case of the wearable sensor, there are only values that are the output, and they need to be reasonably accurate too so that by using these values, the doctor can make decisions. The sensors play a vital role in conveying the patient’s health to the doctor in the remote healthcare monitoring system. The sensors need to be dependable.

#### 6.1.8. Quality of Service

Maintaining the quality of service (QoS) in IoMT is one of its biggest challenges. The devices, cloud computing platforms, sensors, and the existing healthcare management systems (HMS) tend to be extremely heterogeneous. This makes it difficult to integrate them and to measure their QoS. A lack of a standardized methods for service-to-service quality is also felt in hospitals. Another hurdle in QoS is the required data and error tolerance. The delay of a few seconds or an error of a single byte can lead to a life and death situation.

To ensure reliable, fast, and usable IoT devices, we need to improve upon the service quality. Energy constraints, traffic load, and data redundancy are all major challenges faced in the industry for ensuring QoS. To determine QoS for real-time healthcare applications, the QoS criteria are defined as [88]:

QoS = f (cloud QoS, network QoS, location, battery……, N)(1)

N represents the total number of QoS parameters. The parameter is carefully selected. Solving this analytical hierarchy process (AHP) is often used to evaluate different network and cloud parameters based on QoS evaluation criteria [89].

### 6.2. Future Opportunities

**Figure 14:**
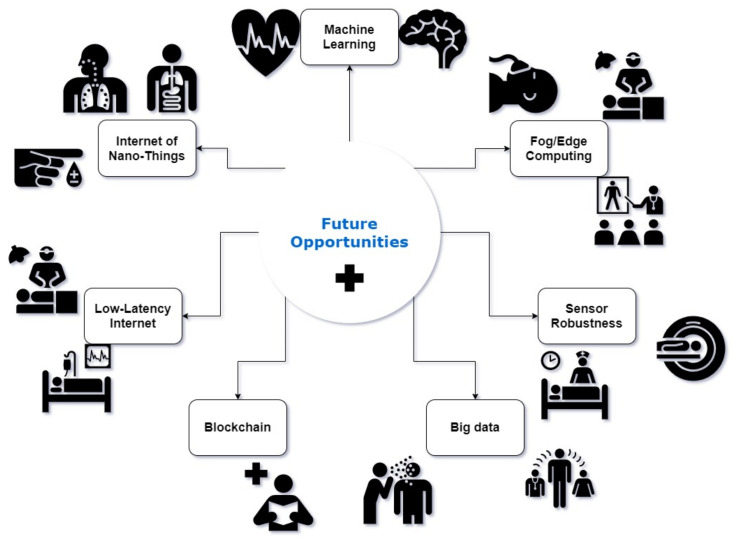
Future opportunities—IoT-assisted wearable sensor systems in healthcare.

The future of IoT and wearables is infinite. Many possibilities are viable and can improve the current system to a considerable extent. The research was mainly based on making a healthcare monitoring system capable of managing and monitoring a certain number of patients in a convenient manner. Moreover, most have successfully implemented it quite gracefully. However, there is always room for the system. Now, the patients are monitored using less calibrated non-medical sensors for the system. There is room to make the medical-grade sensors that are more calibrated and give specific readings and to incorporate various other technology and algorithms to make this HCMS more sophisticated but straightforward at the same time. For simplicity, we have divided the future opportunity section roughly into different domains with room for advances shortly. The main points are highlighted in Figure 14 given below.

#### 6.2.1. Machine Learning

Machine learning has a part to play in taking this society forward. The possibilities of using machine learning are infinite. The market is now full of machine learning algorithms such as artificial neural networks, logistics regressions, discriminant analysis, naive Bayes, etc. These algorithms can be used on the data collected by the sensors to predict different diseases caused by the patient in the near potential future. Nowadays, the real-time prediction of epileptic seizures and strokes is also possible using machine learning algorithms. For this, the sensors must record the patients’ vitals, such as the brain impulses, using an electroencephalogram to record the brain’s electrical potentials and predict the timing of the strokes.

Many papers have also discussed the topic of developing telehealth [42,47,77,90] or e-health [9,53,57], etc. They have paid attention to developing a system that advises the patients on medication by checking their vitals. In these cases, the use of advanced machine learning and artificial intelligence can play a significant role. In the future of telemedicine or telehealth, they can be more precise in detecting the diseases and could be operated on without a doctor or any support staff to supervise it. Integrating this system into the patient’s daily life can help him/her take proper medication without any hassle. This system can be integrated with the intelligent devices that we use, such as smart phones, etc., so that the elderly patients are reminded of the regular check-ups and the daily medicines [91].

Few research works cover the machine learning concept in the healthcare industry, thus creating room for future researchers to dig into the topic and obtain the best out of machine learning.

#### 6.2.2. Fog/Edge

Earlier the use of fog and edge computing has proven to reduce latency in various IoT applications. However, most of this use of IoT fog and edge computing was done in a field other than healthcare. In healthcare, fog and edge computing can create latency benchmarks and a reliable monitoring system for real-time applications [92,93,94].

In the healthcare department, some cases do not seek medical attention but seek the patient’s rehabilitation. In such scenarios, fog and edge computing can be helped using the augmented reality for rehabilitation and trauma therapy. For example, if a patient has a broken leg and he or she cannot walk properly, using a prosthetic leg for the patient and using the VR or AR headsets and trying to walk him or her on a treadmill would be much easier for the patient as well as the doctors to monitor his or her health. The AR and VR headsets can be used to recreate an augmented reality background for the patient so that he or she has the confidence to move around in the actual world without feeling remorse about his or her disability.

#### 6.2.3. Sensor Robustness

The sensors used can be made more robust and used dynamically, at least to partially detect the diseases or any reading, e.g., the pulse sensor is used to detect the pulse rate of the person, but it not only serves to detect the pulse but also has a part to play in detecting the heart rate. Thus, the heart rate can be estimated using the pulse rate sensor, but precise heart rate detection is not performed in this scene. It is calculated by monitoring the blood flow, which can be inaccurate sometimes. This inability of the sensors should be corrected so that it is more reliable. Thus, there is the need to create future of integrating various sensors to obtain both values more accurately to the extent that the heart problems could be detected using a pulse sensor [81,82,95,96].

#### 6.2.4. Big Data

When a critical patient is admitted to the hospital, there are many sensors attached to this patient so that the vitals are appropriately recorded and they show an accurate picture of the patient’s status to the doctor. Moreover, all the sensors work in real-time and record the patients’ vital signs per second, which creates a lot of data. As a result, extensive data analysis comes into the picture for such a scenario.

The extensive data analysis can summarise patients’ history whenever he or she visits the hospital even for a minor cold or in case of any emergency. It creates a much easier way to record the data about the patient and his or her history. The doctors often need to check the patient’s history to decide what medication to be given to the patient in the current circumstances. This system is being used in many of the big hospital chains around India. They created a centralized server that banks all the patients’ data that have visited the hospital by alerting them to a card with a magnetic stripe or a unique card number. All the data of patient’s histories were recorded.

However, there is a massive potential in using these data for patience automatically using extensive data analysis. There is still a possibility to research the e-health system that tracks the patient’s history and uses machine learning algorithms to prescribe medicines and other required drugs.

#### 6.2.5. Blockchain

In recent years, the names blockchain and cryptocurrency are being used and are on the headlines in this pandemic situation for a long time. This concept of blockchain is used to secure cryptocurrency. This property of blockchain can provide extra security to the healthcare databases around the country, which can pave the way for the rise of more secure IoT-based healthcare systems. As we know, IoT and security do not go hand in hand; therefore, the inclusion of blockchain in this sector can prove beneficial for IoT and IoMT. It means the data can be safe and assures that the data are not misused in any manner.

Blockchain makes smart contract-based service subscriptions to provide a more secure and reliable system for healthcare management shortly. The concept of blockchain can enable various companies to provide such a system using their resources more efficiently without worrying about the security of the data collected.

#### 6.2.6. Low-Latency Internet

Low-latency Internet has created many opportunities in the healthcare system which are yet to be exploited. Using such an Internet service can make remote surgeries very reliable Such a low-latency Internet has the potential to make several healthcare applications more trustworthy. Take, for example, remote surgery, which requires a high-precision machine and excellent tactile Internet service with very low latency. This technology is deployed when the doctors are unavailable. Using such a system enables the doctor to perform remote surgery on any patient from where such technology is used. It can also perform minor surgeries on the patients in smaller villages where there are no good hospitals and highly trained medical staff.

Tremor suppression, sensory prosthetics, trauma rehabilitation, interactive medical training, augmented reality, and virtual reality precision in vivo procedures can be possible using low-latency Internet. In India, this point might be easy to implement as there is cheap internet everywhere. Upon installing the 5G network in India, all these services can be enabled and brought into daily use for the healthcare system.

#### 6.2.7. Internet of Nano Things

Internet of Nano Things is a subdivision of the Internet of Medical Things, using small programmable robots that can be remotely controlled or fully automated to perform complex surgeries while staying inside the person’s body. Such robots have the potential to revolutionize the way intricate surgeries are performed. It also makes the current surgical processes minimally invasive.

The current healthcare monitoring system uses external sensors that record and send patients’ vitals to the cloud, such as blood pressure, respiration rate, oxygen level, and many more. Using nano sensors that can be injected or implanted on the person’s body records all of these parameters mentioned above without causing any hindrance for the patient. There is also room for research in this sector to provide an automated nanorobot that can be injected into the person’s body and monitor for any potential diseases. Such a robot would be able to produce a detailed history of the patient’s day-to-day activities with a very high precision that can be helpful for the doctors to take the medical decisions with utmost care and accuracy.

The nano things technology can also be used to fabricate drugs, making them more specific. There is ongoing research on precision medicine. Nowadays, all drugs have some side effects. Precision medicine is the future of pharmacy.

## 7. Conclusions

The paper is a detailed compilation of the evolving technology of IoT in healthcare. It discusses how IoT has changed and connected various industries over the past few decades and has brought the healthcare industry to be more accessible. It starts by giving us an outlook of an IoT-based wearable sensor system for healthcare monitoring. The paper also discusses the comparison of around 133 papers on IoT in healthcare for further improvements in healthcare. It summarizes the sensors used, the focus of research in those papers, and the contribution to the field. We believe this compilation will be a footnote for future research and help us make healthcare accessible to more people. After consulting all of these papers, we could draw a few conclusions on the basic architecture of the IoT-assisted wearable sensor system [97,98,99].

Sensors, communication, cloud services, and data processing and analysis are necessary layers of the architecture to implement IoT in healthcare. It is followed by a comprehensive discussion of data collection, data transfer, data processing, and computing paradigms. The data collection and transfer are part of the patient’s physical layer, usually a wearable system, whereas the storage, computation, and processing are a virtual system which makes accessibility very easy. It gives us an insight into the various computing methods—parallel, cluster, grid, edge, fog, and cloud computing and their workings. The paper covers all the various technology that has been used for communication of the data collected to the server and medical personnel. The technologies include ZigBee, Wi-Fi, Bluetooth, and LoRaWAN. These are the most common short- and long-range communication technologies that have been used in an IoT-based healthcare system. These technologies have been discussed in detail regarding the speed of data transfer, range of communication, power consumption, type of networking, and the various devices available to implement said technology. A tabulation of the technologies in terms of the factors mentioned above and frequency bandwidth, payload, and security is compared [100,101,102,103]. It gives us a better understanding of the type of technology to use depending on the system. Interoperability is discussed, and the necessity of privacy and security in implementing such technologies is also probed. When dealing with private data such as health parameters, privacy must be maintained for the sake of the patient, and with wireless technologies, it is easy to manipulate the data, which may become fatal to the patient instead. Therefore, privacy is an essential aspect of such technologies and needs to be monitored when implementing IoT systems in healthcare. The paper also discusses the application of such IoT wearable sensor systems for healthcare in detail with the various research that has been further with their help. These applications benefit from the real-time monitoring system that is possible with IoT systems and the ability to foreshadow possible abnormalities and severe complications that might arise in at-risk patients. It also eliminates human error or the possibility of overlooking indicators. These systems all give medical personnel the complete picture to make the best decision for the patient and ensure they cure [104,105,106,107].

The review discusses in detail a total of 11 applications of IoT systems in healthcare. Activity recognition is a widespread application of IoT, which is used by almost everyone nowadays. It helps them track their health on their own and keep themselves healthy. It also helps in the case of unfortunate falling or slips in elderly patients who need constant monitoring. The same goes for monitoring the elderly for heart diseases, which requires constant oversight and immediate response in case of any issues. Diabetes is one of the most common diseases in patients worldwide, ranging over a large age group [108,109,110,111].

Moreover, with IoT devices, monitoring critical patients has developed exponentially with accurate data collection. Cardiac patient monitoring has benefited greatly using IoT technology, monitoring various body parameters to anticipate any abnormalities. The same goes for respiratory monitoring, using sensors that continuously calculate the patient’s respiratory function and record it. Sleep monitoring is one application that has become possible with the use of IoT devices in healthcare. It uses multifunctional sensors to help monitor sleep with other vitals to keep the person healthy. Blood pressure monitoring has developed largely, given that almost every fifth person was suffering from BP [112,113,114,115,116].

Moreover, it is something that the majority of the population takes lightly, given that it does not affect them in any drastic way. However, a person’s blood pressure indicates their physical and mental wellness, and with the help of an IoT system, individuals can keep track of their blood pressure. Another important field that progressed with the help of IoT systems is medication adherence. It means making sure that the patient follows the instructions of the doctor. It is highly impossible to track every patient and confirm if they are following their medical regimen. Sometimes missing a few rounds of medications can lead to unnecessary complications, and it can be prevented by helping patients adhere to their prescription. Alzheimer’s disease monitoring has greatly benefited from the use of IoT devices. People with Alzheimer’s must be constantly monitored because they suffer from memory loss and often get lost [117,118,119,120].

IoT system has helped monitor cancer patients, take their treatment progress, and constantly monitor whether the tumor is growing back. The paper also discusses all the commonly used wearable sensors used in IoT systems for healthcare monitoring. Activity detection sensors are very commonly found nowadays, which help users track their health on their own and follow a healthy lifestyle. Respiratory sensors are critical. They track the patients’ blood oxygen level and the breathing rate of the patients who are unconscious and recovering from complex surgeries. Heartbeat sensors help us record any data from the heart that can help us anticipate many healthcare issues. An unhealthy heart can indicate many issues in a person. Blood pressure sensors help us monitor the user’s general health and indicate a healthy lifestyle. Blood pressure can help anticipate many issues and is often overlooked. As mentioned earlier, diabetes is a severe ailment, and glucose monitoring sensors help us monitor the blood glucose level of diabetic people constantly. The temperature sensor is a very commonly found sensor, a very primitive indicator of ailment in a person. Fluctuation in body temperature usually indicates trouble. A tabulation of the sensors and their application, sensing parameter, wearable type, and characteristics is detailed [121,122,123,124].

The papers dealing with different types of sensors are indicated in the table to give a comprehensive idea of the existing systems of IoT in healthcare. This is followed by a brief exposure to various technologies and systems that are currently found in healthcare, and it gives us an idea of what is available currently and how it can be developed to make healthcare services easily accessible. A description of what CE and FDA approval is and why it is necessary is also covered. It moves on to include the quality of service (QoS) of these technologies and devices. The paper next discusses open problems that we face with IoT systems in healthcare that need to be addressed to implement them on a larger scale. The most common problems are the collected data range and the sensing capabilities of the sensor, along with its size. Since it has to be worn by the user, it needs to be of small size, which might affect the other parameters of the sensor. The next important problem to address is power consumption. Given that the system would be wearable, we need to optimize the power consumption not to require frequent charging. That defeats the purpose of the system. The most important to address is the privacy of the data collected, since it can be misused and can become fatal to the patient in the wrong hands. Moreover, for IoT to be implemented on a large scale, it must address and derive a solution. Another problem we face with wearable technology is its wearability, because it needs to fit people and not be obvious and blend with the current fashion. Even though it is not a priority, this still plays an important role in implementing IoT in healthcare. Safety is another issue we need to make sure is attended to, and we cannot have the same sensors that were supposed to help with the health of the user affect their health. Thus, the long-term effects of these wearables must be tested. In addition, currently, we have many IoT technologies that are not in regulation to medical requirements [125,126,127,128].

The review is a very detailed compilation of IoT wearable technology in the healthcare industry and covers all the advantages and issues we face with the technology. With the data recorded here, we can develop a well-perfected system without flaws and implement infinite possibilities depending on the patients and the requirements. Future scope includes a physical implementation of such a system with live data collection, analysis, and computation. We can explore different communication technologies and security options available and record the performance. We can develop customized sensor arrays that can make wearability easy and less obvious. We can also implement ML to the system to make data processing faster and more accurate [129,130,131]. We can also implement blockchain to bring a layer of security needed in the system to perform to its capabilities [132,133].

## Figures and Tables

**Table 1:** Comparison with previous surveys.

Year	IoT Wearable Sensors	Health Focus	Contributions of Existing Surveys	Ref.
2018	Body sensors	Glucose, heart rate, blood pressure, body temperature	An intelligent healthcare network using IoThNet topology is discussed.	[10]
2018	SPO2 sensor, BP sensor, EKG sensor, EMG sensor, Motion sensor, Medical super sensor	-	The paper deals with a medical cyber-physical system, networked medical device systems, and IT-based services, emerging medical systems	[11]
2018		Focuses on storing, privacy, and validity of the data that comes through a wearable sensor	This survey provides a comparison of various uses and methods in cloud computing, fog computing, IoT, and embedded systems in healthcare monitoring, interactive healthcare challenges, and the changes that big data analytics has brought on.	[1]
2019	BAN sensors used. Smartwatch sensing the ECG, EMG, and EEG		Survey dedicated to the healthcare monitoring system advancements specifically for chronically patients and the elderly. This includes the environmental sensing around the patients and the measure to detect chronic heart failures.	[12]
2019	-	-	The paper discusses the implementation of ML in resource-scarce embedded systems.	[13]
2019	Smartwatch, smart contact lenses, intelligent asthma management, ingestible sensors, inhalers, activity trackers	EHR, pills, consultation with doctors, overall fitness, health, and healthcare	The survey reviews all the existing devices and systems available and gives a brief overview and function.	[14]
2020	HCMS, e-health	Focuses on monitoring patients accurately. No particular disease stated	Surveys are about the overview of the current tech in the IOTM and the sensors and actuators that can help develop a superior HCMS.	[9]
2020	Blockchain		Survey to point out the usage of blockchain in securing the IoT data.	[4]
2021	-	-	A table is used to summarize that a combination of ML/DL with healthcare IoT and Cloud can be used to solve various security threats	[15]

**Table 2:** Wireless technologies comparison for wearable communication.

	Characteristics							Ref.
Type	Topology	Frequency Bands	Range	Data Rate	Power Consumption	Payload	Security	
ZigBee	Star, ad hoc, and mesh	2 GHz (global)	10 to 100 m	250 Kbps	low	68 bytes	AES block cipher	[38]
LoRaWAN	Star	169 MHz (Asia), 868 MHz (Europe) 91 MHz (North America	15 to 20 km	250 bps to 5.5 kbps	low	51 bytes	unique 128-bit AES key and a globally unique identifier (EUI-64-based DevEUI)	[39]
Wi-Fi	Point to hub	2.4 GHz, 5 GHz	10 to 100 m	6.75 Gbps	high	A Wi-Fi packet is about 2312 bytes	RC4 stream cipher AES, WPA4	[36]
Bluetooth	Point to point, point to multipoint	Between 2.402 GHz to 2.408 GHz	10 to 100 m	2.1 Mbps	high	251 bytes	Basic	[40]

**Table 3:** Various IoT-assisted Wearable Sensors for Healthcare Monitoring.

SNo.	Application	Sensor	Characteristics	Sensed Parameter	Wearable Type	Ref
1.	Heartbeat Monitoring	ECG; AD8232; MAXL335cc	Inexpensive, Obtrusive	Heartrate	Wristband	[28,53,58,59]
2.	Temperature	LM35; DHT11	Inexpensive, noninvasive	Body temperature	Wristband	[3,4,5,19,22,24,58,59,60,61,62,63,64,65]
3.	Glucose monitoring	Glucose sensor; INA219	Invasive, expensive	Blood glucose	Patch on arm/strip	[6,42,45,46,53
4.	Respiratory	Pulse Oximeter	Inexpensive, easy to use	Blood oxygen saturation	Clamp on finger	[3,19,45,46,53,54,66,67,68]
5.	Respiratory	Airflow sensor	Obtrusive	Breathing rate	Worn on face	[33,51]
6.	GSR	GSR sensor	Expensive, noninvasive	Sweat gland activity	Patch on arm	[46]
7.	Acceleration	Acceleration sensor; ADXL345	Inexpensive, noninvasive	Movement	Wristband	[58]
8.	Breathing	MQ2 sensor	Inexpensive, noninvasive	Acetone in Breadth	Mouth piece	[34]
9.	Load	Strain Gauge load cell	Inexpensive, noninvasive	Weight of medicine	Medicine box	[69]
10.	Communication	GSM module, Wi-Fi module	Storage, Backup	Transferring data	Wristband	[5,6,20,28,29,31,34,51,62,64,65,70,71,72,73]
11.	Touch	Pressure sensor	Non-invasive, Expensive	Pressure on skin	Patch on skin	[74]
12.	Moisture	Moisture sensor	Non-expensive	Moisture	Wristband	[71]
13.	Organizing	RFID sensor	Non-expensive	RF waves	Tag	[3,5,21,23,24,59,75,76,77,78,79]
14.	Movement	PIR	Non-expensive, Not attached to the body	IR rays	Attached to fixed body	[23]
15.	Touch	GSR sensor	Expensive, nonintrusive	Sweat glands	Patch on skin	[46]

## Data Availability

Not applicable.

## Abbreviations

IoT	Internet Of Things
HMS	Healthcare Monitoring System
WSN	Wireless Sensor Network
BAN	Body Area Network
GPS	Global Positioning System
WSN	Wireless Sensor Management
MCU	Microcontroller Unit
BP/BG/BT/HB	Blood Pressure/Blood Glucose/Body Temperature/Heartbeat
EHMS	E-Healthcare Monitoring System
IOMT	Internet of Medical things
ECG/EKG	Electrocardiogram
EHR	Electronic Health record
ML/DL	Machine Learning/Deep Learning
HCMS	Healthcare Monitoring System
EMG	Electromyography
EEG	Electroencephalogram
RFID	Radio Frequency Identification
WPAN	Wireless Personal Area Network
LPWAN	Low-Power Wide Area Network
FSK	Frequency Shift Keying
CSS	Chirp Spread Spectrum
BMI	Body Mass Index
WBSN	Wireless Body Sensor Network/Wearable Body Sensor Network
AR/VR	Augmented reality/Virtual reality
TSST	Trier Social Stress Test
